# Effects of Different Green Teas on Obesity and Non-Alcoholic Fatty Liver Disease Induced by a High-Fat Diet in Mice

**DOI:** 10.3389/fnut.2022.929210

**Published:** 2022-06-24

**Authors:** Dan-Dan Zhou, Qian-Qian Mao, Bang-Yan Li, Adila Saimaiti, Si-Yu Huang, Ruo-Gu Xiong, Ao Shang, Min Luo, Hang-Yu Li, Ren-You Gan, Hua-Bin Li, Sha Li

**Affiliations:** ^1^Guangdong Provincial Key Laboratory of Food, Nutrition and Health, Department of Nutrition, School of Public Health, Sun Yat-sen University, Guangzhou, China; ^2^Research Center for Plants and Human Health, Institute of Urban Agriculture, Chinese Academy of Agricultural Sciences, Chengdu, China; ^3^School of Public Health, Shanghai Jiao Tong University School of Medicine, Shanghai, China

**Keywords:** green tea, obesity, non-alcoholic fatty liver disease, prevention, antioxidant activity

## Abstract

Non-alcoholic fatty liver disease (NAFLD) and obesity are serious public health problems. Green tea is widely consumed in the world and different green teas could possess different bioactivities. In this study, the effects of 10 selected green teas on obesity and NAFLD were evaluated and compared. The mice fed with a high-fat diet were intervened with green tea extract (200 mg/kg body weight) for 15 weeks. Most of these teas were first evaluated for their effects on obesity and NAFLD. The results showed that Selenium-Enriched Chaoqing Green Tea and Jieyang Chaoqing Tea showed the most prominent inhibition of obesity and body weight gains of mice in these two tea intervention groups and model groups were 5.3, 5.5, and 13.7 g, respectively. In addition, Jieyang Chaoqing Tea, Taiping Houkui Tea, and Selenium-Enriched Chaoqing Green Tea exerted the most notable effect on NAFLD, which was attributed to decreasing body weight, and lipid content and ameliorating oxidative stress. Furthermore, 13 phytochemicals were determined in these teas by high-performance liquid chromatography and the correlation analysis found that epigallocatechin gallate, gallocatechin, and epigallocatechin might contribute to the decrease of hepatic weight, while epicatechin might reduce oxidative stress. In general, several green teas could prevent the development of obesity and NAFLD and could be developed into functional foods. This study was also helpful for the public to select appropriate tea to prevent obesity and NAFLD.

## Introduction

Non-alcoholic fatty liver disease (NAFLD) is a threatening chronic non-communicable disease with continuously increased incidence worldwide. The prevalence of NAFLD is estimated at 25% among adults, 3–10% in the pediatric population, and could rise to 70% among children with obesity (1–3). Various factors contribute to the development of NAFLD. Increasing evidence revealed that obesity is a strong contributor to the occurrence of NAFLD and obesity itself is also a serious public health problem. In the condition of similar body weight, people with more visceral fat are more likely to develop NAFLD (4–6). The lipid metabolism relied on the action of the liver, such as the *de-novo* lipogenesis, lipid oxidation, and uptake/secretion of lipoproteins. When excessive intake of dietary fat and energy exceeds the capacity of the liver to metabolize lipids, the synthesis and deposition of a lipid would increase in the liver and body, leading to lipid metabolic disturbance, which is of great importance in the pathogenesis of NAFLD. The increase in the level of free fatty acids is accompanied by the overload of lipids, which could promote the over generation of reactive oxygen species (ROS) and the consumption of antioxidants. Oxidative stress could further impair the function of hepatocytes and even destroy the hepatic structure, which would accelerate the process of NAFLD (7, 8). NAFLD could develop into more severe liver diseases, such as fibrosis, cirrhosis, and hepatocellular carcinoma (9, 10). Therefore, prevention and treatment of NAFLD are vital. On the other hand, some natural products have shown strong antiobesity and/or antioxidant activity, which could be a good alternative for the prevention of NAFLD and obesity because of the limited efficacy and potential side effects of chemically synthetic drugs.

Tea (*Camellia sinensis*) is a popular drink with a long history. It has shown various bioactivities, such as antioxidant, anti-inflammation, antidiabetic, antiobesity, anticancer, and cardiovascular protection (11–15). Tea could be divided into green, white, yellow, oolong, black, and dark teas based on different fermentation degrees. Green tea extract has been used to treat antiobesity (11). On the other hand, growing studies showed the effect of green tea on the prevention and management of NAFLD (16–18). The hepatoprotective and antiobesity effects of green tea are mainly attributed to its rich bioactive compounds, such as polyphenols. However, different kinds of green teas could have very different compositions and contents of bioactive compounds. Hence, the effects of different kinds of green teas on NAFLD and obesity could be different. In this study, the effects of 10 selected green teas on obesity and NAFLD induced by a high-fat diet (HFD) were evaluated and compared in mice at a dose of 200 mg/kg body weight (bw). Most of these green teas were first evaluated for their effects on obesity and NAFLD. The findings could serve the public to choose tea for the prevention of obesity and NAFLD. In addition, several teas could be developed into functional food for the prevention and management of obesity and NAFLD.

## Materials and Methods

### Chemicals and Materials

Methanol, formic acid, and isopropanol were obtained from Macklin Chemical Factory (Shanghai, China). The standard chemicals were offered by Derick Biotechnology Corporation Ltd. (Chengdu, China), namely, gallic acid, quercitrin, kaempferol, astragalin, quercetin, ellagic acid, theaflavin, myricetin, chlorogenic acid (CA), caffeine, catechin, epicatechin (EC), gallocatechin (GC), catechin gallate (CG), epigallocatechin (EGC), epicatechin gallate (ECG), gallocatechin gallate (GCG), and epigallocatechin gallate (EGCG). The determination kits of hepatic triglyceride (TG) and malondialdehyde (MDA) were provided by Apply-gen Technologies Corporation Ltd. (Beijing, China) and kits for superoxide dismutase (SOD) and glutathione (GSH) were obtained from Nanjing Jiancheng Bioengineering Institute (Nanjing, China). Moreover, the contents of serum TG, total cholesterol (TC), low-density lipoprotein cholesterol (LDL-C), aspartate transaminase (AST), and alanine transaminase (ALT) were determined by kits from Roche Diagnostics (Shanghai, China).

The details of 10 selected green teas are shown in Table 1.

**Table 1:** The details of 10 selected green teas from China.

**No**.	**Name**	**Production place**
GT1	Dianqing Tea	Kunming city, Yunnan province
GT2	Jieyang Chaoqing Tea	Jieyang city, Guangdong province
GT3	Fenggang Zinc-Selenium-Enriched Tea	Guiyang city, Guizhou province
GT4	Liping Xiang Tea	Liping city, Guizhou province
GT5	Taiping Houkui Tea	Huangshan city, Anhui province
GT6	Xihu Longjing Tea	Hangzhou city, Zhejiang province
GT7	Chaoqing Green Tea	Yichang city, Hubei province
GT8	Selenium-Enriched Chaoqing Green Tea	Enshi city, Hubei province
GT9	Selenium-Enriched Matcha	Enshi city, Hubei province
GT10	Seven Star Matcha	Shaoxing city, Zhejiang province

### Preparation of Green Tea Extracts

The preparation of 10 kinds of green tea extracts was carried out according to the literature (19). The 10 g green tea sample and 100 ml boiling deionized water were mixed in a conical flask and extracted in a 98°C water bath for 10 min. The mixture was filtered to collect the infusion. The tea sample was extracted 3 times and the infusions were collected and merged. Then, the collected infusion was concentrated using a vacuum rotary evaporator at 60°C and about 15 ml of concentrated infusion was obtained. Finally, the concentrated infusion was freeze-dried into powder by a lyophilizer and kept at −80°C. The powders were dissolved in deionized water to obtain the tea extracts with a concentration of 20 g/l (w/v) before the administration to the mice.

### Animal Study

The C57BL/6J male mice (18–20 g) used in this study were purchased from the Experimental Animal Center of Guangdong Province (Guangzhou, China). All the mice were housed in a specific pathogen-free (SPF) animal laboratory, where the humidity was set at 40–60%, the room temperature was 22 ± 0.5°C, and the light/dark cycle was 12 h. After acclimatization for 1 week, 8-week-old mice were randomly divided into the normal diet (ND) control group, the high-fat diet (HFD) model group, and the green tea (GT) treatment groups (including 10 different green tea treatment groups), totally 12 groups (*n* = 10/group). The ND control group was fed with a normal diet with an energy of 3.6 kcal/g (12% calories from fat), which was provided by Jiangsu Xietong Pharmaceutical Bioengineering Corporation Ltd. (Nanjing, China). Meanwhile, the HFD model group and the treatment group were given a high-fat diet with the energy of 5.0 kcal/g (60% calories from fat), which was purchased from Trophic Animal Feed High-technology Corporation Ltd. (Nantong, China). Mice in the treatment groups were intragastrically administrated with tea extracts at a dose of 200 mg/kg bw daily for 15 weeks according to the literature (20, 21). In addition, mice in the control and model groups were received 10 ml/kg bw of deionized water by gavage because the concentration of the intervention solution was 20 g/l, which was equal to 10 ml/kg bw when a dose of 200 mg/kg bw was used. The daily food consumption and weekly body weight of mice were recorded. At the end of the 15-week intervention, all the mice were fasted for 12 h and then weighed, anesthetized, and sacrificed to collect blood and liver samples, and perirenal and epididymal adipose tissues. All the experimental procedures involving animals in this study have received approval from the Ethics Committee in the School of Public Health, Sun Yat-Sen University (No. 2019–002; 28 February 2019).

### Measurement of Serum Alanine Transaminase, Aspartate Transaminase, Triglyceride, Total Cholesterol, and Low-Density Lipoprotein Cholesterol Levels

After being collected and placed at room temperature (25°C) for 1 h, the blood samples were centrifuged at 3,000 × *g* (4°C) for 15 min to acquire serum. The levels of serum ALT, AST, TG, TC, and LDL-C were determined with the instruction of detection kits. In brief, enzymatic tests were used to measure the contents of TG, TC, and LDL-C and the velocity tests were carried out to determine the levels of AST and ALT.

### Biochemical Analysis of Hepatic Tissue

The determination of hepatic TG content was performed according to the instruction from the detection kit. The 25 mg hepatic tissue was completely homogenized with 500 μl lysis buffer to obtain the liver homogenate. After placing for 10 min, the upper layer of liver homogenate was separated and heated at 70°C for 10 min and then centrifuged (2,000 × *g*, 25°C) for 5 min to obtain the supernatant to measure the TG content.

The status of hepatic oxidative stress in mice was assessed by the determination of GSH, MDA, and SOD levels. The 200 mg hepatic tissue was mixed and homogenized with 1.8 ml of 0.9% NaCl to acquire hepatic homogenate. The homogenate was centrifuged (2,500 × *g*, 4°C) for 10 min to get the supernatant for the determination of GSH and SOD. In addition, 10 mg hepatic tissue was mixed and homogenized with 500 μl lysis buffer to obtain liver homogenate, which was then centrifuged (10,000 × *g*, 4°C) for 10 min to obtain the supernatant to measure MDA content.

### Observation of Histopathological Changes in Liver and Adipose Tissues

The histopathological changes in liver and epididymal adipose tissues were examined using H&E staining. The hepatic and epididymal adipose tissues were soaked in 4% paraformaldehyde for a few days and then embedded in paraffin. The embedded samples were sliced into 5-μm-thick sections and then deparaffinized, rehydrated, and stained with H&E. The image of the liver and epididymal adipose tissue was captured and observed with a microscope.

### Measurement of Bioactive Compounds in Green Tea Extracts

The bioactive components in green tea extracts were qualitative and quantitative analyzed using high-performance liquid chromatography (HPLC) in comparison with the standard compounds, which is according to our previous study (19).

### Statistical Analysis

The analysis of experimental data was performed with the software SPSS version 25.0 (IBM Incorporation, Armonk, NY, USA) and the results were expressed as mean ± SD. A one-way ANOVA combined with the least significant difference (LSD) test was conducted to compare the difference between the experimental groups. Statistical significance was set at *p* < 0.05. In addition, the software GraphPad Prism 8 (GraphPad Software, La Jolla, CA, USA) was applied to draw figures.

## Results and Discussion

### Effects of Different Green Teas on Body Weight

**Figure 1:**
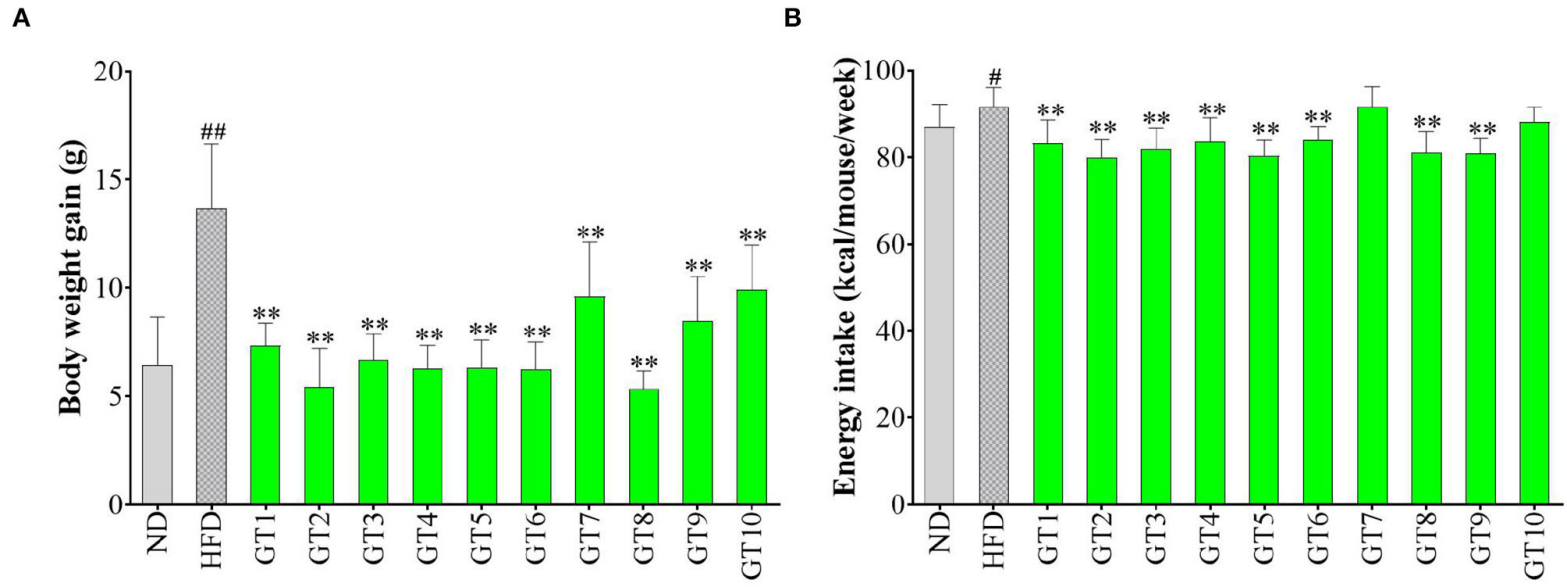
Effects of green teas on body weight (BW) gain **(A)** and energy intake **(B)**. # *p* < 0.05, ## *p* < 0.01, the model group vs. the control group; ** *p* < 0.01, the green tea groups vs. the model group. ND, normal diet; HFD, high-fat diet. GT1, Dianqing Tea; GT2, Jieyang Chaoqing Tea; GT3, Fenggang Zinc-Selenium-Enriched Tea; GT4, Liping Xiang Tea; GT5, Taiping Houkui Tea; GT6, Xihu Longjing Tea; GT7, Chaoqing Green Tea; GT8, Selenium-Enriched Chaoqing Green Tea; GT9, Selenium-Enriched Matcha; GT10, Seven Star Matcha.

The effects of 10 kinds of green teas on body weight gain and energy intake are shown in Figure 1. As shown in Figure 1A, mice fed with HFD had a significantly larger weight gain than those of the control group (*p* < 0.01). The average body weight gain of mice in the model group was 13.7 g, which was 2.14-fold that in the control group (6.4 g). In addition, the body weight gain in all the green tea groups was markedly smaller than that in the model group (*p* < 0.01), but the effect greatly varied in different green teas. The Selenium-Enriched Chaoqing Green Tea (GT8) and Jieyang Chaoqing Tea (GT2) showed the strongest inhibition on obesity with 5.3 and 5.5 g body weight gain, respectively. Moreover, the energy intake in the model group was significantly bigger than that in the control group (*p* < 0.05). The energy intake in mice treated with 8 green teas was reduced compared with the model group (*p* < 0.01) (Figure 1B). The results of energy intake were generally coincident with those of body weight gain, which manifested that green tea played a role in the inhibition of obesity partly because of its effect on the reduction of energy intake.

A large amount of research has proved that excessive and frequent consumption of a high-calorie diet led to the prevalence of obesity and related NAFLD. In this study, HFD induced a dramatic increase in body weight gain in C57BL/6 mice, while green tea could effectively prevent obesity at a dose of 200 mg/kg bw. The results were consistent with previous studies (16, 20, 22). For example, a study found that the oral gavage with 500 mg/kg bw of green tea extracts markedly attenuated body weight gain in C57BL/6 mice compared with the HFD group (16). Weight loss is usually accompanied by a decrease in energy intake. In this study, most of the 10 green teas significantly inhibited the energy intake, except Chaoqing Green Tea (GT7) and Seven Star Matcha (GT10). Correspondingly, the body weight gain in mice treated with Chaoqing Green Tea (GT7) and Seven Star Matcha (GT10) was more obvious than in other teas. Hence, the antiobesity effects of 10 different green teas varied greatly, which was partly due to the inhibitory difference in energy intake.

### Effects of Green Teas on Visceral Adipose Tissue

**Figure 2:**
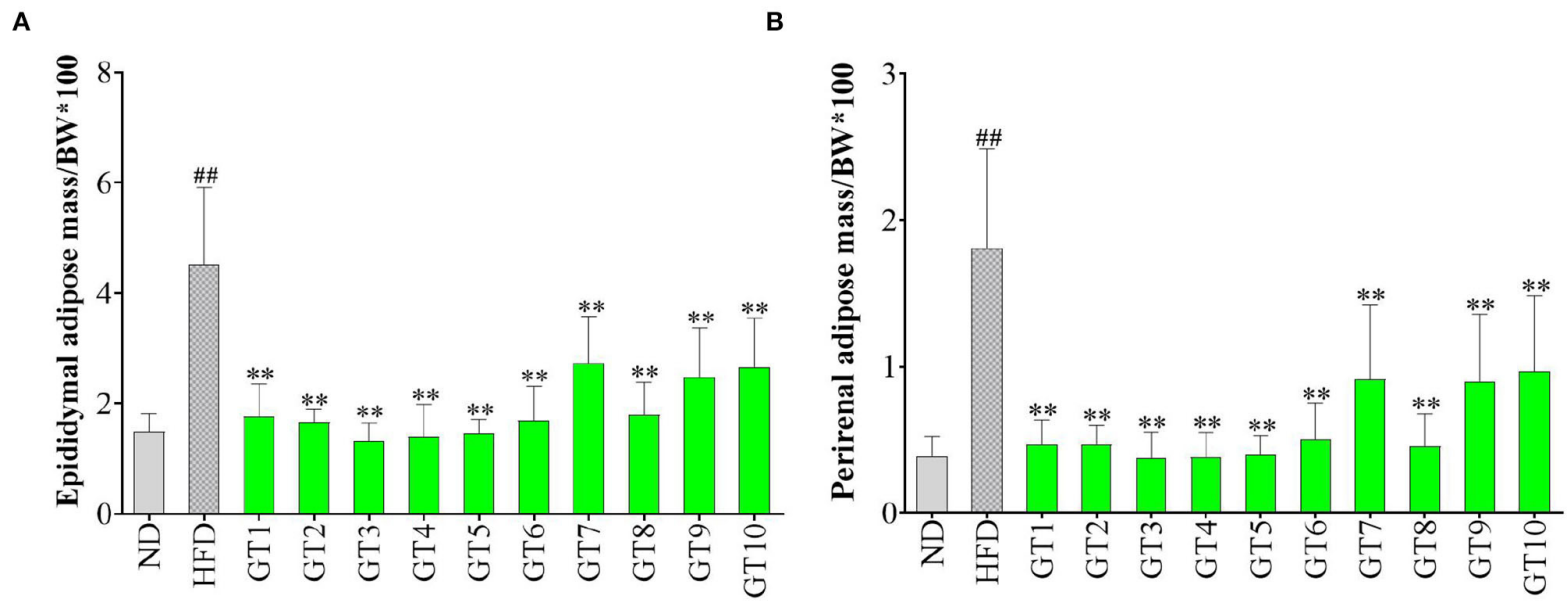
Effects of green teas on visceral adipose tissue. **(A)** Epididymal adipose mass/BW × 100 and **(B)** Perirenal adipose mass/BW × 100. ## *p* < 0.01, the model group vs. the control group; ** *p* < 0.01, the green tea groups vs. the model group. BW, body weight; ND, normal diet; HFD, high-fat diet; GT1, Dianqing Tea; GT2, Jieyang Chaoqing Tea; GT3, Fenggang Zinc-Selenium-Enriched Tea; GT4, Liping Xiang Tea; GT5, Taiping Houkui Tea; GT6, Xihu Longjing Tea; GT7, Chaoqing Green Tea; GT8, Selenium-Enriched Chaoqing Green Tea; GT9, Selenium-Enriched Matcha; GT10, Seven Star Matcha.

**Figure 3:**
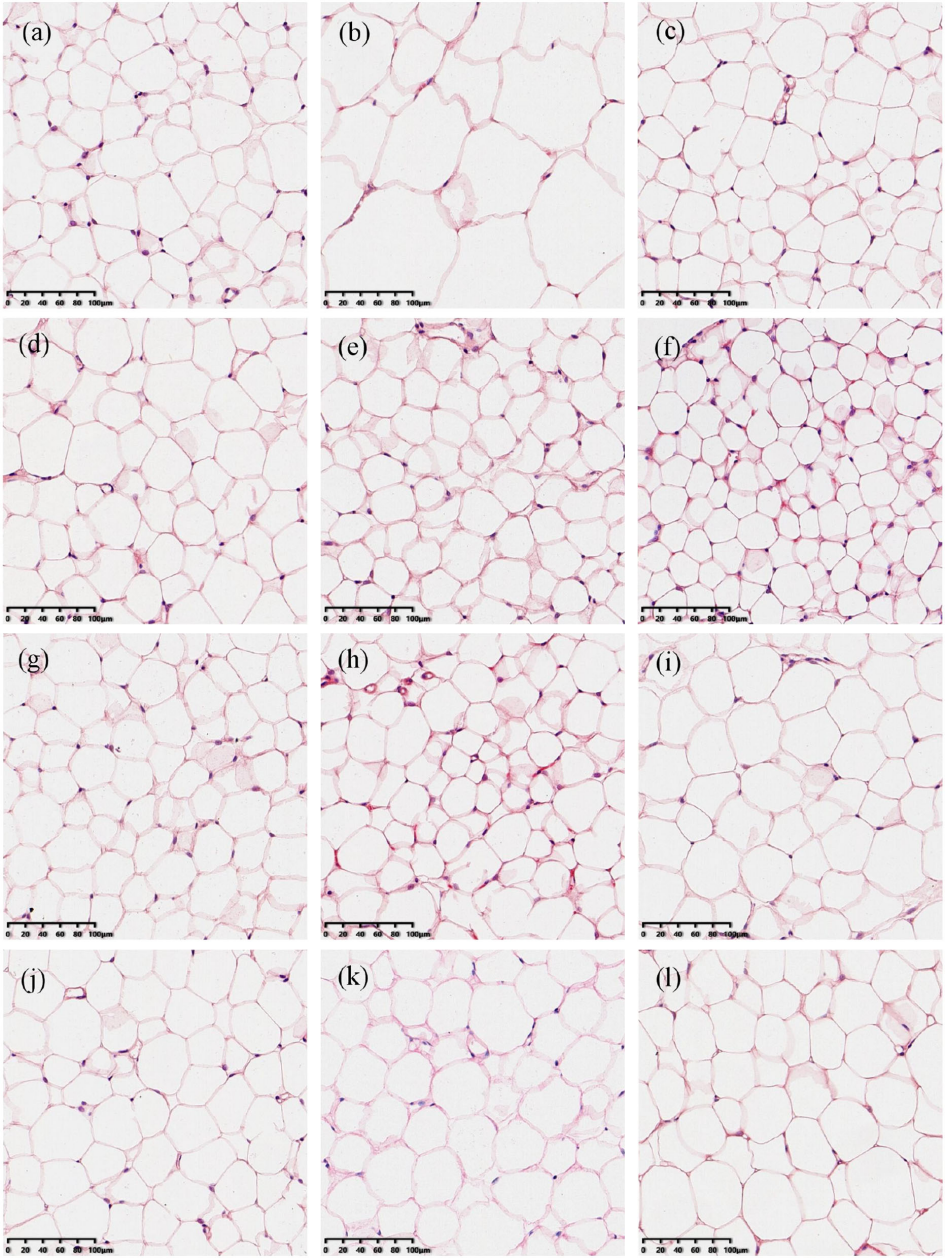
Histopathological changes of epididymal adipose tissues (200X magnification). **(a)** The control group; **(b)** the model group; **(c)** the GT1 group; **(d)** the GT2 group; **(e)** the GT3 group; **(f)** the GT4 group; **(g)** the GT5 group; **(h)** the GT6 group; **(i)** the GT7 group; **(j)** the GT8 group; **(k)** the GT9 group; and **(l)** the GT10 group. ND, normal diet; HFD, high-fat diet; GT1, Dianqing Tea; GT2, Jieyang Chaoqing Tea; GT3, Fenggang Zinc-Selenium-Enriched Tea; GT4, Liping Xiang Tea; GT5, Taiping Houkui Tea; GT6, Xihu Longjing Tea; GT7, Chaoqing Green Tea; GT8, Selenium-Enriched Chaoqing Green Tea; GT9, Selenium-Enriched Matcha; GT10, Seven Star Matcha.

Epididymal and perirenal adipose is the important visceral fat in the body. The ratio of their mass to body weight was used to evaluate the effect of green teas on visceral adipose in this study and the results are shown in Figure 2. The percentages of both the epididymal and perirenal adipose masses in the body weight of mice in the model group significantly increased compared with those of the control group (*p* < 0.01), while the treatment of different green teas could reduce the accumulation of visceral fat in mice induced by HFD at varying degree. Among 10 kinds of green teas, Fenggang Zinc-Selenium-Enriched Tea (GT3), Liping Xiang Tea (GT4), and Taiping Houkui Tea (GT5) showed the most remarkable effectiveness in suppressing the increase of visceral adipose tissue. Furthermore, the histopathological changes in epididymal adipose tissue have been studied and the results are shown in Figure 3. It could be seen that the sizes of epididymal adipocytes in the model group had an incredible expansion and the shapes of adipocytes showed an abnormal irregularity. On the other hand, epididymal adipocytes in the different green tea groups were significantly smaller and had more regular shapes compared with the model group.

Increasing evidence revealed that the accumulation of visceral adipose tissue was a strong predictor of the occurrence of NAFLD. In the case of similar body weight, people with a higher proportion of visceral fat were more prone to develop NAFLD (23, 24). In this study, the visceral adipose tissues in the model group markedly increased and the morphology of epididymal adipose tissue was abnormal mainly characterized by the expansive sizes and irregular shapes of adipocytes. These adverse changes were ameliorated by the intervention of green tea. Some studies also revealed the effect of green tea on inhibiting the accumulation of visceral fat. In a previous study, the visceral adipose mass of obese mice and lean littermates fed with diets containing 1 or 2% green tea extract was significantly lower than their respective controls fed with a green tea extract-free diet (25). In another study, an HFD significantly increased the weight of epididymal and perirenal adipose tissues, and the size of adipocytes in mice, while the dietary supplement Matcha prevented these unfavorable changes (26). Likewise, Selenium-Enriched Matcha (GT9) and Seven Star Matcha (GT10) in this study could reduce the accumulation of adipose and the hypertrophy of adipocytes. In addition, we found that several other green teas had better effects on visceral adipose tissues than the Matcha teas, such as Fenggang Zinc-Selenium-Enriched Tea and Liping Xiang Tea.

### Effects of Green Teas on Hepatic Weight and Triglyceride Content of Liver

**Figure 4:**
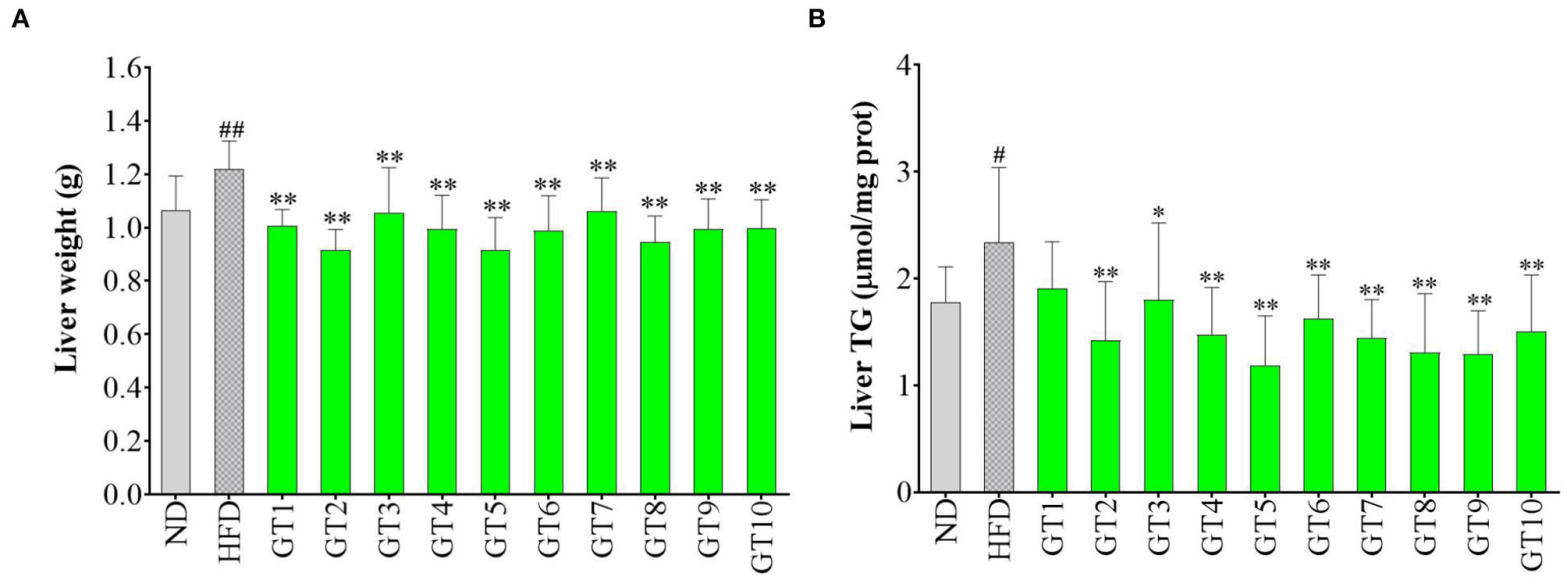
Effects of green teas on weight and TG content of liver. **(A)** Liver weight and **(B)** Liver triglyceride (TG) content. # *p* < 0.05, ## *p* < 0.01, the model group vs. the control group; * *p* < 0.05, ** *p* < 0.01, the green tea groups vs. the model group. ND, normal diet; HFD, high-fat diet; GT1, Dianqing Tea; GT2, Jieyang Chaoqing Tea; GT3, Fenggang Zinc-Selenium-Enriched Tea; GT4, Liping Xiang Tea; GT5, Taiping Houkui Tea; GT6, Xihu Longjing Tea; GT7, Chaoqing Green Tea; GT8, Selenium-Enriched Chaoqing Green Tea; GT9, Selenium-Enriched Matcha; GT10, Seven Star Matcha.

In this study, the hepatic weight and TG content of the liver in mice fed with HFD markedly increased compared with mice fed with a normal diet (*p* < 0.05) (Figure 4). All the 10 green teas could significantly decrease the hepatic weight (*p* < 0.01), while the effect varied in different teas (Figure 4A). Jieyang Chaoqing Tea (GT2), Taiping Houkui Tea (GT5), and Selenium-Enriched Chaoqing Green Tea (GT8) exerted a relatively notable effect on decreasing the hepatic weight. Accordingly, the majority of 10 green teas, except Dianqing Tea (GT1), could reduce the content of hepatic TG (*p* < 0.05) (Figure 4B). The most effective teas of decreasing TG content were Taiping Houkui Tea (GT5), followed by Selenium-Enriched Matcha (GT9) and Selenium-Enriched Chaoqing Green Tea (GT8).

The hepatic weight and TG content of liver were important predictors of hepatic lipid accumulation and the hepatic steatosis (25). The effects of green teas on hepatic weight and TG content in this study were in line with previous studies (16, 22, 27). For example, a study pointed out that the dietary supplement of 1% green tea extract decreased hepatic weight and TG content in obese mice (27).

### Effects of Green Tea on Aspartate Transaminase and Alanine Transaminase Levels

**Figure 5:**
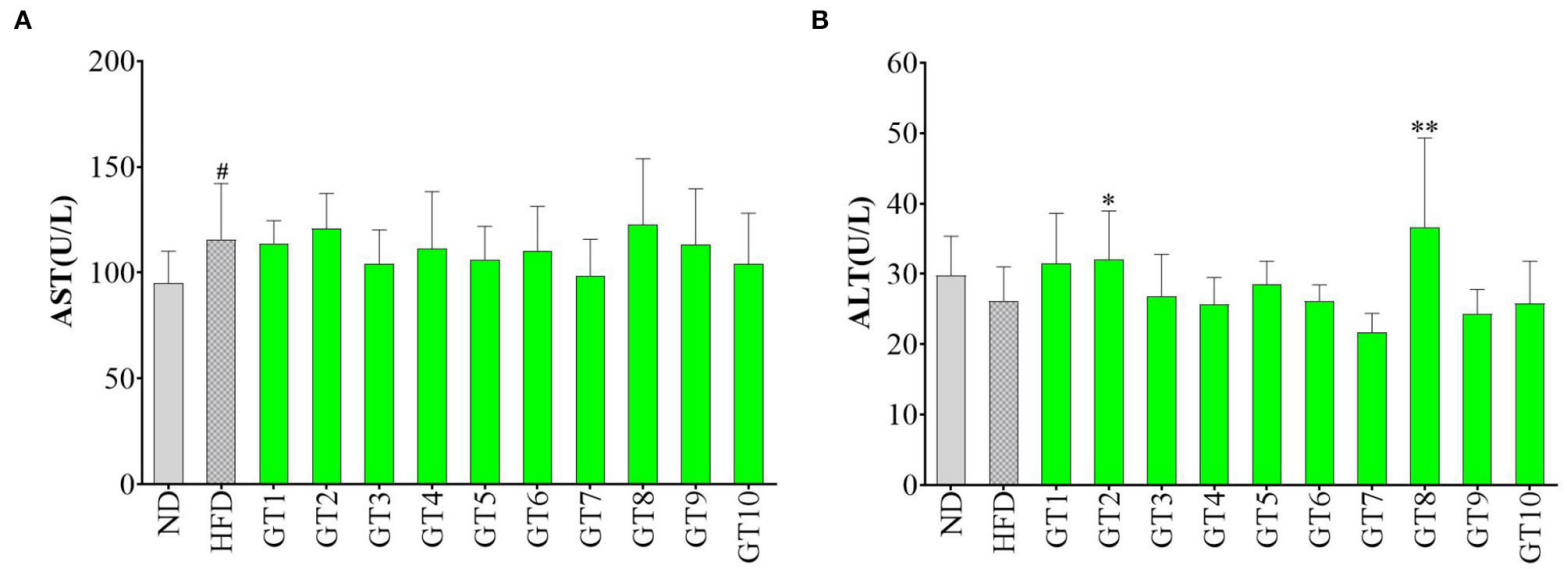
Effects of green teas on liver injury. **(A)** Serum AST level and **(B)** Serum ALT level. # *p* < 0.05, the model group vs. the control group; * *p* < 0.05, ** *p* < 0.01, the green tea groups vs. the model group. AST, aspartate transaminase; ALT, alanine aminotransferase; ND, normal diet; HFD, high-fat diet; GT1, Dianqing Tea; GT2, Jieyang Chaoqing Tea; GT3, Fenggang Zinc-Selenium-Enriched Tea; GT4, Liping Xiang Tea; GT5, Taiping Houkui Tea; GT6, Xihu Longjing Tea; GT7, Chaoqing Green Tea; GT8, Selenium-Enriched Chaoqing Green Tea; GT9, Selenium-Enriched Matcha; GT10, Seven Star Matcha.

The liver injury was accessed by the detection of serum AST and ALT levels and results are shown in Figure 5. In comparison with the control group, although no significant increment of ALT level was observed in the model group (*p* > 0.05) (Figure 5B), AST level in serum was remarkably elevated (*p* < 0.05) (Figure 5A), hinting the existence of liver injury induced by HFD. No significant difference in AST and ALT levels was observed between most of the 10 green teas groups and model group, but a decreasing trend of AST and ALT levels was found in the Fenggang Zinc-Selenium-Enriched Tea (GT3) and Chaoqing Green Tea (GT7) groups (*p* > 0.05). On the other hand, Jieyang Chaoqing Tea (GT2) and Selenium-Enriched Chaoqing Green Tea (GT8) could further increase the ALT activity (*p* < 0.05), which hinted their possible damage to the hepatocytes in mice.

The hepatocytes were gradually destroyed by various factors induced by HFD, leading to the liver injury and NAFLD. The increase of serum ALT and AST activities was sensitive indicator in response to the liver injury. A previous study found that the oral gavage with green tea extract (500 mg/kg bw) for 12 weeks could prevent the increase of serum ALT and AST activities in male C57Bl/6 mice fed with HFD and protect against liver injury (16). Another study showed that green tea intervention reversed the increase of serum ALT activity induced by HFD (28). In this study, 10 green teas did not remarkably decrease the activities of these two transaminases, which could be because the dosage of intervention was smaller (200 mg/kg bw) than that in the literature (500 mg/kg bw) (16).

### Histopathological Evaluation of Liver

**Figure 6:**
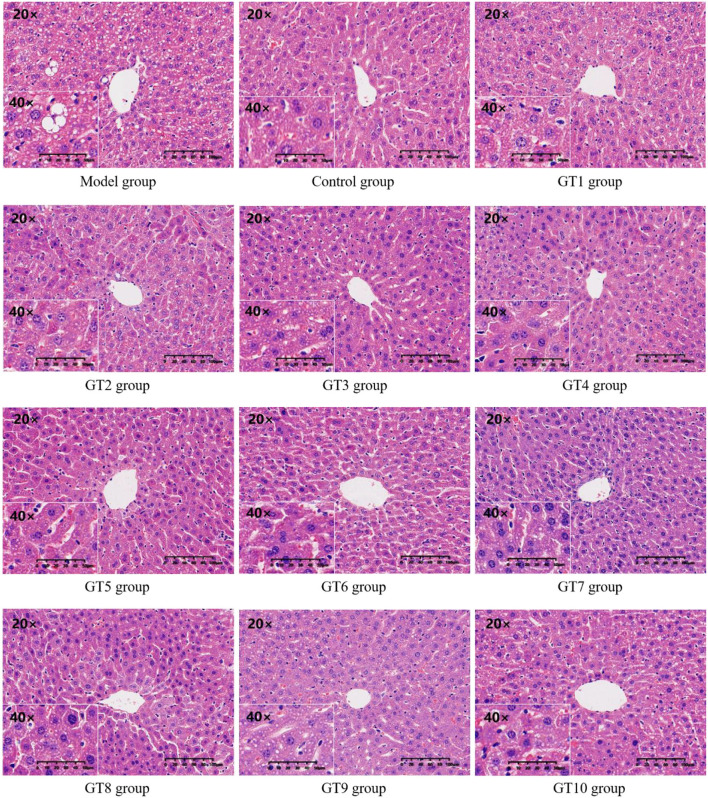
The histopathological images of hepatic tissue (200 and 400X magnification). GT1, Dianqing Tea; GT2, Jieyang Chaoqing Tea; GT3, Fenggang Zinc-Selenium-Enriched Tea; GT4, Liping Xiang Tea; GT5, Taiping Houkui Tea; GT6, Xihu Longjing Tea; GT7, Chaoqing Green Tea; GT8, Selenium-Enriched Chaoqing Green Tea; GT9, Selenium-Enriched Matcha; GT10, Seven Star Matcha.

The H&E staining was performed to observe the histopathological changes of liver induced by a HFD and to further verify the effect of green teas on NAFLD. The results are given in Figure 6. Compared with the control group, many unequal size lipid droplets presented in the hepatocytes of the model group. Of note, lipid droplets in hepatocytes were significantly reduced by the intervention of green teas, particularly in Fenggang Zinc-Selenium-Enriched Tea (GT3), Liping Xiang Tea (GT4), Chaoqing Green Tea (GT7), and Selenium-Enriched Chaoqing Green Tea (GT8). The results were consistent with previous studies (16, 17, 20).

### Effects of Green Teas on Serum Lipid Levels

**Figure 7:**
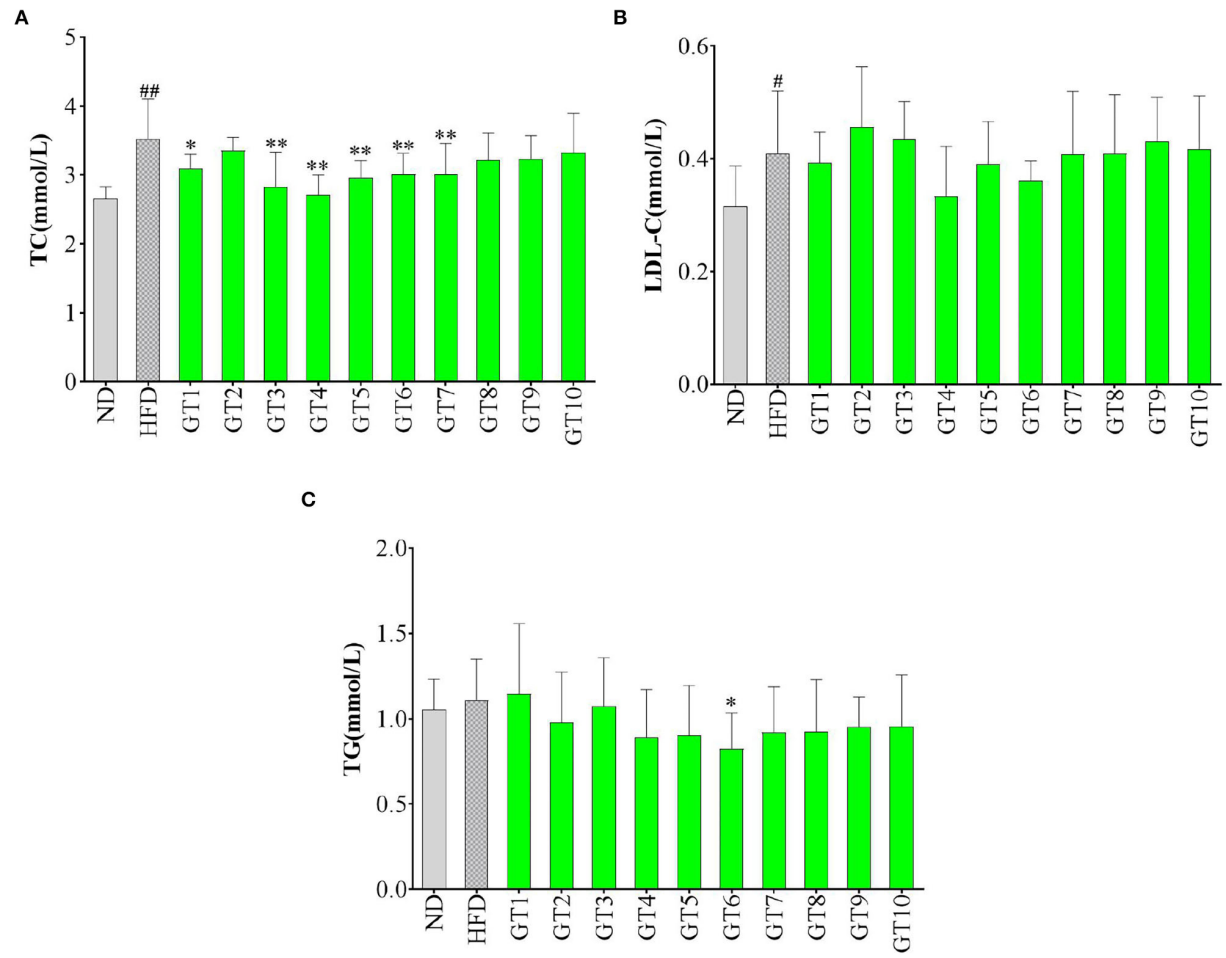
Effects of green teas on serum lipid levels. **(A)** serum total cholesterol (TC) level; **(B)** serum low-density lipoprotein cholesterol (LDL-C) level; and **(C)** serum TG level. # *p* < 0.05, ## *p* < 0.01, the model group vs. the control group; * *p* < 0.05, ** *p* < 0.01, the green tea group vs. the model group. GT1, Dianqing Tea; GT2, Jieyang Chaoqing Tea; GT3, Fenggang Zinc-Selenium-Enriched Tea; GT4, Liping Xiang Tea; GT5, Taiping Houkui Tea; GT6, Xihu Longjing Tea; GT7, Chaoqing Green Tea; GT8, Selenium-Enriched Chaoqing Green Tea; GT9, Selenium-Enriched Matcha; GT10, Seven Star Matcha.

The serum TC, TG, and LDL-C levels have been used to evaluate the lipid levels in this study and the results are shown in Figure 7. Compared with the control group, mice fed with HFD had markedly higher levels of serum TC and LDL-C (*p* < 0.05), which reflected the presence of hyperlipidemia. The effects of 10 green teas on lipid levels varied greatly and some green teas showed prominent effectiveness in decreasing lipid levels. The 6 out of 10 green teas showed significant effect in lowering TC levels, namely, Liping Xiang Tea (GT4), Fenggang Zinc-Selenium-Enriched Tea (GT3), Taiping Houkui Tea (GT5), Xihu Longjing Tea (GT6), Chaoqing Green Tea (GT7), and Dianqing Tea (GT1) (Figure 7A). Although there was no significant difference existed in LDL-C level between the model group and the 10 green tea groups (*p* > 0.05), a decreasing tendency was observed in the Liping Xiang Tea (GT4) and Xihu Longjing Tea (GT6) groups (Figure 7B). On the other hand, TG content in mice fed with a HFD was not evidently elevated compared with that in mice fed with normal diet (*p* > 0.05). In addition, there was no significant difference in TG level between the model group and the majority of the 10 green tea groups (*p* > 0.05), except the Xihu Longjing Tea (GT6) group obviously decreased the level of TG (*p* < 0.05) (Figure 7C).

Lipid metabolism disorder is one of the most prominent manifestations of NAFLD, which could be presented as hyperlipidemia, hypertriglyceridemia, and hypercholesterolemia (29, 30). The TC, TG, and LDL-C contents in serum were the most commonly used indicators to reflect the lipid profiles (21, 31). Several studies showed that green tea could ameliorating the development of NFLDA *via* decreasing the contents of serum lipids and improving the lipid profile (20, 25, 27, 32). In this study, different green teas exerted different effects on lipid indicators. From a comprehensive perspective, Xihu Longjing Tea (GT6) and Liping Xiang Tea (GT4) could be the most efficient green teas to improve lipid profile.

### Effects of Green Teas on Hepatic Oxidative Stress

**Figure 8:**
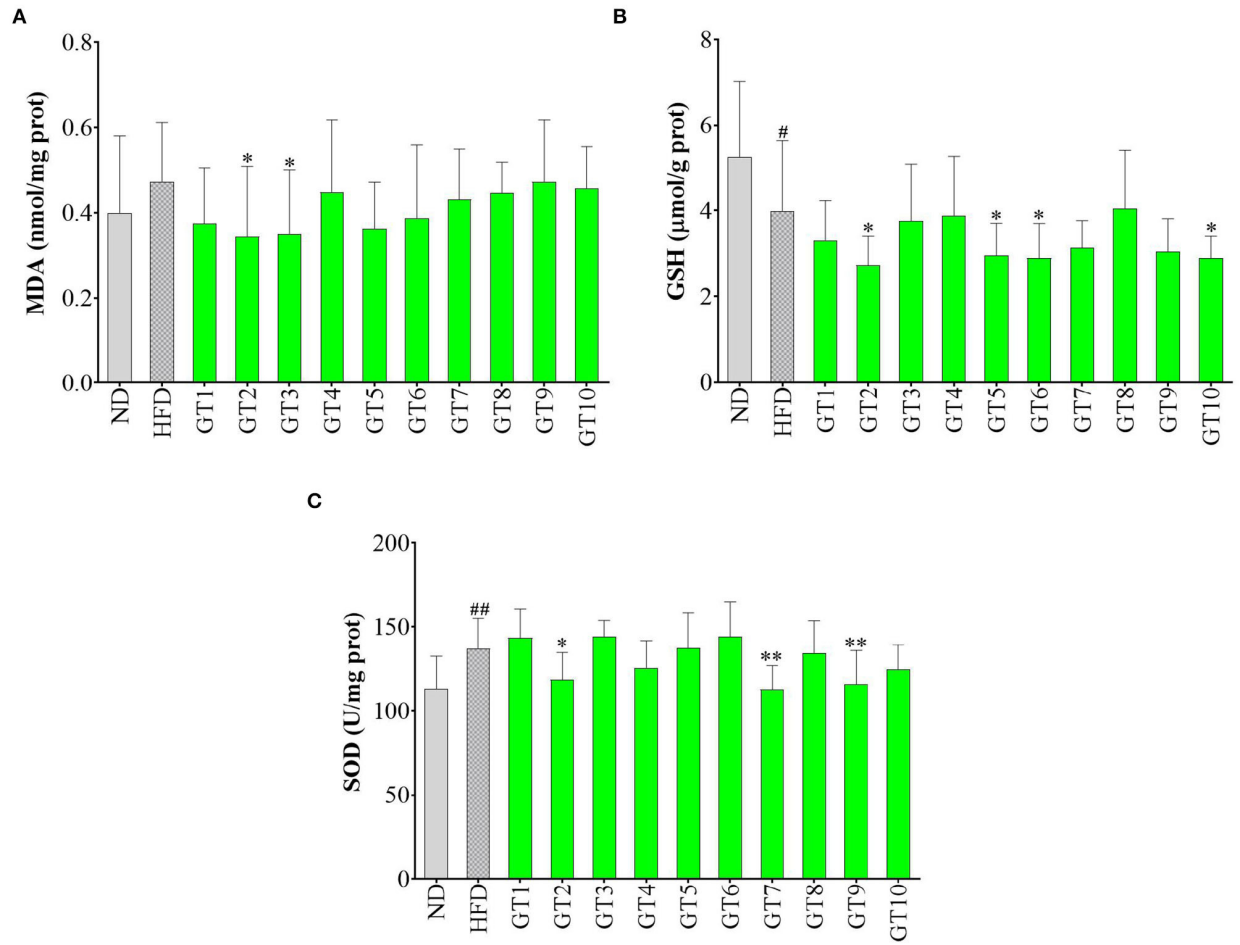
Effects of green teas on hepatic oxidative stress. **(A)** Glutathione (GSH) (μmol/g protein); **(B)** superoxide dismutase (SOD) (U/mg protein); and **(C)** malondialdehyde (MDA) (nmol/mg protein). # *p* < 0.05, ## *p* < 0.01, the model group vs. the control group; * *p* < 0.05, ** *p* < 0.01, the green tea groups vs. the model group. GT1, Dianqing Tea; GT2, Jieyang Chaoqing Tea; GT3, Fenggang Zinc-Selenium-Enriched Tea; GT4, Liping Xiang Tea; GT5, Taiping Houkui Tea; GT6, Xihu Longjing Tea; GT7, Chaoqing Green Tea; GT8, Selenium-Enriched Chaoqing Green Tea; GT9, Selenium-Enriched Matcha; GT10, Seven Star Matcha.

In this study, the levels of hepatic MDA, GSH, and SOD were detected to reflect the redox state in the liver and the results are shown in Figure 8. As shown in Figure 8, the model group showed a lower content of GSH and an increasing tendency of MDA content in contrast with the control group, indicating that oxidative stress occurred in the model group. In addition, the Jieyang Chaoqing Tea (GT2) and Fenggang Zinc-Selenium-Enriched Tea (GT3) groups showed a significant decrease of the MDA content compared with the model group (*p* < 0.05), suggesting their antioxidant activity (Figure 8A).

Oxidative stress is regarded as a crucial contributor to the occurrence and development of NAFLD. With the accumulation and oxidation of fatty acids in the liver, the antioxidants are consumed and excessive reactive oxygen species are produced, gradually destroying the structure and function of hepatocytes and eventually accelerating the process of NAFLD (33, 34). MDA is a representative product of lipid peroxidation (35). This study found that HFD led to an escalating trend of MDA level in mice, while some green teas, namely, Jieyang Chaoqing Tea (GT2) and Fenggang Zinc-Selenium-Enriched Tea (GT3), could significantly lower the MDA level, hence inhibiting the lipid peroxidation and oxidative stress. The results were consistent with some previous report. For example, in an ob/ob mice Non-alcoholic steatohepatitis model, diet supplementation with 0.5 and 1% green tea extract for 6 weeks inhibited the generation of ROS, along with the reduction of lipid peroxidation (36). SOD is an important antioxidative enzyme and GSH is a non-enzymatic antioxidants (10). The overgeneration of ROS caused the consumption of antioxidant substances, subsequently might led to the decrease of GSH content and SOD activity. However, in this study, several green teas could lower the levels of GSH and SOD, which was contrary to the expectation. This could be because the concentration of antioxidants in these teas was too high and they would show pro-oxidant activity in the body, just as vitamin C, which is a strong antioxidant *in vitro* and will be pro-oxidant under high concentration *in vivo* (37, 38).

### Bioactive Components in Green Tea Extracts

**Figure 9:**
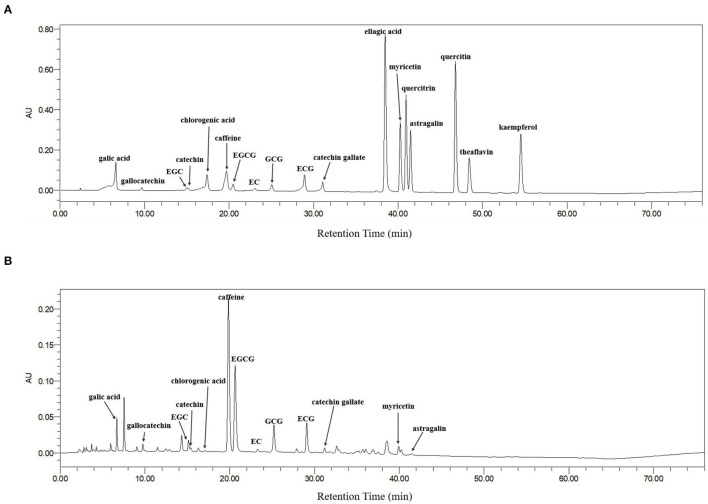
The high-performance liquid chromatography (HPLC) chromatograms of the standard components **(A)** and Liping Xiang Tea **(B)** at 254 nm.

Bioactive components in green tea extracts were identified by HPLC *via* comparison with standard compounds. The chromatograms of the standard substances and Liping Xiang Tea are given in Figure 9 and the contents of major phytochemicals in 10 green teas are shown in Table 2. In general, 8 types of catechins and 5 other active compounds (caffeine, gallic acid, chlorogenic acid, astragalin, and myricetin) were detected and quantified in green tea extracts (Figure 9). The results showed that EGCG, epigallocatechin (EGC), epicatechin, caffeine, and gallic acid could be detected in 10 green teas and most green teas contained gallocatechin gallate (GCG), epicatechin gallate (ECG), catechin, gallocatechin, catechin gallate, chlorogenic acid, and astragalin. Myricetin was only found in Dianqing Tea (GT1), Liping Xiang Tea (GT4), and Xihu Longjing Tea (GT6).

**Table 2:** The contents of major phytochemicals in 10 green tea extracts (mg/g DW).

**Phytochemicals**	**GT1**	**GT2**	**GT3**	**GT4**	**GT5**
EGCG	237.64 ± 2.77	262.32 ± 5.81	157.91 ± 5.00	248.37 ± 4.34	266.45 ± 7.40
GCG	50.24 ± 0.86	103.66 ± 1.52	40.15 ± 1.81	54.66 ± 0.96	77.75 ± 1.70
ECG	40.77 ± 0.43	16.83 ± 0.23	28.85 ± 0.70	24.02 ± 0.35	26.02 ± 0.29
EGC	31.22 ± 0.28	107.48 ± 3.49	37.87 ± 0.91	26.77 ± 0.43	40.99 ± 3.58
Catechin	34.68 ± 0.37	-	38.60 ± 2.15	17.31 ± 0.51	36.38 ± 0.52
Epicatechin	20.11 ± 1.03	30.07 ± 2.09	25.93 ± 0.96	14.48 ± 0.29	17.08 ± 1.05
GC	19.47 ± 1.40	95.36 ± 1.20	28.81 ± 0.59	18.93 ± 0.34	44.27 ± 3.59
CG	8.04 ± 0.27	5.21 ± 0.74	6.46 ± 0.60	6.13 ± 0.28	7.59 ± 0.44
Caffeine	78.11 ± 0.43	154.22 ± 3.00	106.46 ± 0.54	75.29 ± 1.23	107.33 ± 1.11
Gallic acid	8.40 ± 0.09	16.17 ± 0.77	11.52 ± 0.33	8.83 ± 0.40	12.27 ± 0.31
CA	5.18 ± 0.06	-	4.51 ± 0.04	1.69 ± 0.02	-
Astragalin	-	2.05 ± 0.21	5.22 ± 0.31	5.74 ± 0.18	3.16 ± 0.02
Myricetin	1.83 ± 0.06	-	-	4.17 ± 0.14	-
**Phytochemicals**	**GT6**	**GT7**	**GT8**	**GT9**	**GT10**
EGCG	232.93 ± 6.39	162.03 ± 3.75	196.69 ± 1.94	234.29 ± 13.30	201.48 ± 5.72
GCG	63.22 ± 1.34	38.56 ± 1.10	30.95 ± 1.19	-	-
ECG	15.19 ± 0.33	14.68 ± 0.30	9.20 ± 0.10	22.75 ± 0.91	-
EGC	43.65 ± 1.87	18.40 ± 0.26	86.84 ± 1.85	66.37 ± 1.60	46.08 ± 1.61
Catechin	-	-	16.52 ± 0.30	-	53.15 ± 4.53
Epicatechin	12.95 ± 0.41	11.03 ± 0.49	12.82 ± 0.20	19.24 ± 0.68	10.68 ± 0.65
GC	33.69 ± 0.39	16.44 ± 0.24	51.34 ± 0.23	41.31 ± 1.77	-
CG	3.96 ± 0.09	3.55 ± 0.17	2.08 ± 0.09	-	-
Caffeine	88.18 ± 0.17	56.98 ± 0.59	54.40 ± 0.28	110.22 ± 1.25	131.10 ± 0.92
Gallic acid	7.16 ± 0.24	9.49 ± 0.25	2.60 ± 0.11	19.57 ± 0.25	1.91 ± 0.18
CA	2.11 ± 0.02	1.66 ± 0.04	-	2.93 ± 0.01	-
Astragalin	2.65 ± 0.11	-	4.47 ± 0.14	9.62 ± 0.15	-
Myricetin	2.20 ± 0.15	-	-	-	-

*Abbreviations: –, not detected; DW, dry weight; GT1, Dianqing Tea; GT2, Jieyang Chaoqing Tea; GT3, Fenggang Zinc-Selenium-Enriched Tea; GT4, Liping Xiang Tea; GT5, Taiping Houkui Tea; GT6, Xihu Longjing Tea; GT7, Chaoqing Green Tea; GT8, Selenium-Enriched Chaoqing Green Tea; GT9, Selenium-Enriched Matcha; GT10, Seven Star Matcha; EGCG, epigallocatechin gallate; GCG, gallocatechin gallate; ECG, epicatechin gallate; CG, catechin gallate; GC, gallocatechin; EGC, epigallocatechin; CA, chlorogenic acid*.

The contents of 13 phytochemicals in different green teas varied greatly (Table 2). EGCG was the most abundant catechin in green tea extracts (157.91 ± 5.00–266.45 ± 7.40 mg/g DW), followed by GCG (30.95 ± 1.19–103.66 ± 1.52 mg/g DW), EGC (18.40 ± 0.26–107.48 ± 3.49 mg/g DW), gallocatechin (16.44 ± 0.24–95.36 ± 1.20 mg/g DW), catechin (16.52 ± 0.30–53.15 ± 4.53 mg/g DW), ECG (9.20 ± 0.10–40.77 ± 0.43 mg/g DW), epicatechin (10.68 ± 0.65–30.07 ± 2.09 mg/g DW), and catechin gallate (2.08 ± 0.09–8.04 ± 0.27 mg/g DW). The 10 green teas had also a high content of caffeine (54.40 ± 0.28–154.22 ± 3.00 mg/g DW). However, the contents of gallic acid (1.91 ± 0.18–19.57 ± 0.25 mg/g DW), astragalin (2.05 ± 0.21–9.62 ± 0.15 mg/g DW), chlorogenic acid (1.66 ± 0.04–5.18 ± 0.06 mg/g DW), and myricetin (1.83 ± 0.06–4.17 ± 0.14 mg/g DW) were relatively low in green tea extracts.

**Figure 10:**
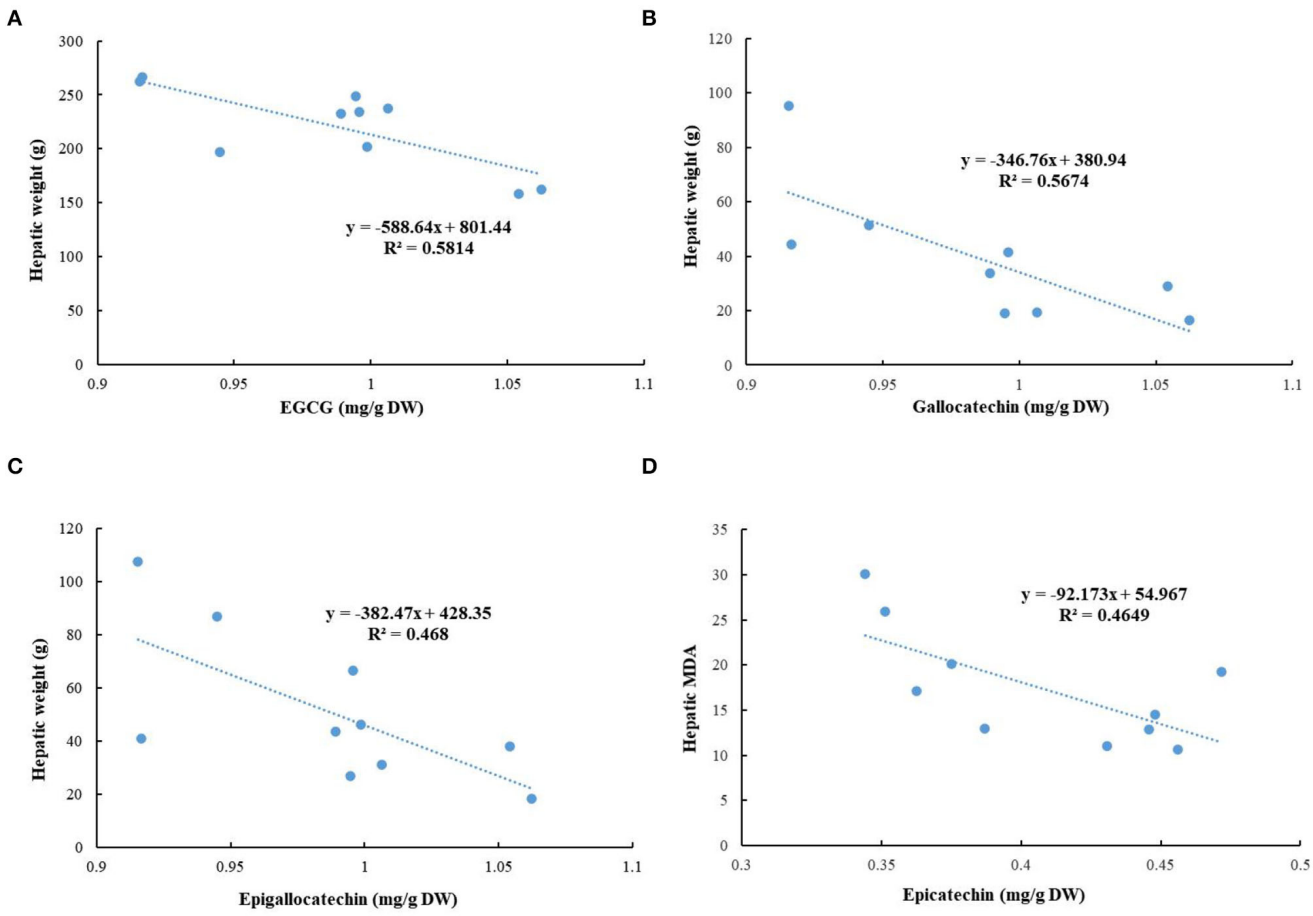
Correlation analysis. **(A)** Epigallocatechin gallate (EGCG) and hepatic weight; **(B)** gallocatechin and hepatic weight; **(C)** epigallocatechin and hepatic weight; and **(D)** epicatechin and hepatic MDA.

The correlation analysis was conducted to evaluate the associations among detected phytochemicals in green tea extracts and the tested biochemical indicators in this study (Figure 10). The negative relationships were existed in the hepatic weight and contents of EGCG (*R*^2^ = 0.5814), gallocatechin (*R*^2^ = 0.5674), and epigallocatechin (*R*^2^ = 0.468), hinting that these compounds might contribute to the decrease of hepatic weight. In addition, a negative association was observed between the content of epicatechin and hepatic MDA (*R*^2^ = 0.4649), suggesting that epicatechin could reduce oxidative stress.

The rich bioactive ingredients in green tea conferred its powerful hepatoprotective and antiobesity effects, especially polyphenols. Catechins are the major phenolic compounds in green tea, such as EGCG, GCG, EGC, ECG, and catechin (39). As the most abundant catechins in green tea, EGCG was most well studied and some studies indicated the preventive properties of EGCG against obesity and NAFLD. An experimental study pointed out that the dietary supplementation with 0.4% EGCG (w/w) for 14 weeks effectively prevented the development of NAFLD induced by a HFD in mice (21). Moreover, the coadministration of EGCG and caffeine exerted a more remarkable effect on NAFLD amelioration than their single use by suppression of body weight gain, and reduction of energy intake and white adipose tissue weight in mice (40). Therefore, green teas with high contents of EGCG and caffeine could be an excellent alternative for the prevention and management of obesity and NAFLD.

## Conclusion

The effects of 10 different green teas on obesity and NAFLD induced by a HFD were evaluated and compared in mice. Although all the 10 green teas showed antiobesity, their effects varied greatly. The Selenium-Enriched Chaoqing Green Tea and Jieyang Chaoqing Tea were the most effective teas in inhibiting body weight gain and the Fenggang Zinc-Selenium-Enriched Tea, Liping Xiang Tea, and Taiping Houkui Tea could markedly inhibit the increase of visceral adipose tissues. It was found that several green teas, such as Jieyang Chaoqing Tea, Taiping Houkui Tea, and Selenium-Enriched Chaoqing Green Tea, could effectively prevent the occurrence of NAFLD and underlying mechanisms were inhibiting body weight gain, reducing the accumulation of visceral fat, improving lipid profile and oxidative stress, and ameliorating hepatic steatosis. Furthermore, 13 phytochemicals in green tea extracts were separated and quantified by an HPLC method and the correlation analysis showed that EGCG, gallocatechin, and epigallocatechin could contribute to the decrease of hepatic weight and epicatechin could reduce oxidative stress. In conclusion, most of 10 green teas were first evaluated for their effects on obesity and NAFLD. Several green teas showed strong effects and could be developed into functional foods to prevent obesity and NAFLD. In addition, if the intervention dose (200 mg/kg bw) for mice in this study was transformed into that for persons, this dose could be obtained by daily drinking tea, indicating the people could prevent obesity and NAFLD by daily drinking some green teas. Therefore, the findings could also serve the public to select suitable tea for the prevention and management of obesity and NAFLD.

## Data Availability Statement

The raw data supporting the conclusions of this article will be made available by the authors, without undue reservation.

## Ethics Statement

All experimental procedures involving animals in this study have received the approval from the Ethics Committee in School of Public Health, Sun Yat-sen University (No. 2019–002; 28 February 2019).

## Author Contributions

D-DZ, Q-QM, SL, R-YG, and H-BL: conceptualization. D-DZ, Q-QM, B-YL, ASa, S-YH, R-GX, ASh, ML, and H-YL: investigation. D-DZ: writing—original draft preparation. SL, R-YG, and H-BL: writing—reviewing and editing. H-BL: funding acquisition. All authors have read and agreed to the published version of the manuscript.

## Funding

This study was supported by the National Key R&D Program of China (No. 2018YFC1604405) and the Key Project of Guangdong Provincial Science and Technology Program (No. 2014B020205002).

## Conflict of Interest

The authors declare that the research was conducted in the absence of any commercial or financial relationships that could be construed as a potential conflict of interest.

## Publisher's Note

All claims expressed in this article are solely those of the authors and do not necessarily represent those of their affiliated organizations, or those of the publisher, the editors and the reviewers. Any product that may be evaluated in this article, or claim that may be made by its manufacturer, is not guaranteed or endorsed by the publisher.
